# Association between human paraoxonase 2 protein and efficacy of acetylcholinesterase inhibiting drugs used against Alzheimer’s disease

**DOI:** 10.1371/journal.pone.0258879

**Published:** 2021-10-29

**Authors:** Fauzia Parween, Md. Summon Hossain, Kshetra Pal Singh, Rinkoo Devi Gupta

**Affiliations:** 1 Faculty of Life Sciences and Biotechnology, South Asian University, New Delhi, India; 2 Defence Research and Development Establishment (DRDO), Gwalior, India; Panjab University Chandigarh, INDIA

## Abstract

Serum Paraoxonase 2 (PON2) level is a potential biomarker owing to its association with a number of pathophysiological conditions such as atherosclerosis and cardiovascular disease. Since cholinergic deficiency is closely linked with Alzheimer’s disease (AD) progression, acetylcholinesterase inhibitors (AChEIs) are the treatment of choice for patients with AD. However, there is a heterogenous response to these drugs and mostly the subjects do not respond to the treatment. Gene polymorphism, the simultaneous occurrence of two or more discontinuous alleles in a population, could be one of the important factors for this. Hence, we hypothesized that PON2 and its polymorphic forms may be hydrolyzing the AChEIs differently, and thus, different patients respond differently. To investigate this, two AChEIs, donepezil hydrochloride (DHC) and pyridostigmine bromide (PB), were selected. Human PON2 wildtype gene and four mutants, two catalytic sites, and two polymorphic sites were cloned, recombinantly expressed, and purified for *in vitro* analysis. Enzyme activity and AChE activity were measured to quantitate the amount of DHC and PB hydrolyzed by the wildtype and the mutant proteins. Herein, PON2 esterase activity and AChE inhibitor efficiency were found to be inversely related. A significant difference in enzyme activity of the catalytic site mutants was observed as compared to the wildtype, and subsequent AChE activity showed that esterase activity of PON2 is responsible for the hydrolysis of DHC and PB. Interestingly, PON2 polymorphic site mutants showed increased esterase activity; therefore, this could be the reason for the ineffectiveness of the drugs. Thus, our data suggested that the esterase activity of PON2 was mainly responsible for the hydrolysis of AChEI, DHC, and PB, and that might be responsible for the variation in individual response to AChEI therapy.

## Introduction

The paraoxonase (PON) family comprises three genes, PON1, PON2, and PON3, which are calcium-bound hydrolases, and are related to several diseases [1, 2]. All three PONs are lactonases and differ in substrate specificities [3, 4]. PON1 is the most studied among the three PONs due to its multifactorial role in organophosphate (OP) metabolism, cardiovascular disease (CVD), and the presence of relevant polymorphisms [5, 6]. PON2 does not have organophosphatase activity like PON1, but is capable of hydrolyzing lactones and several aromatic carboxylic acid esters [7, 8]. Understanding the catalytic mechanism of an enzyme is important in developing it for therapeutic purpose, and also provide insights into the efficacy of prescribing drugs [9, 10].

PON family shares ~60% sequence identity in amino acid sequences [11]. PON2 has shown similarities with PON1 on many grounds [12]. It has antioxidant properties similar to those of PON1 in preventing LDL oxidation [13, 14]. Barathi et al. found a common ligand binding pattern shared by PON1 and PON2 for arylesterase and lactonase activity. PON2 like PON1 has the same residues exhibiting hydrogen bonding interaction for both the activities, and includes the H115 position [15, 16]. Similarly, R/Q polymorphism at position 192 in PON1 modulates the micro-environment of the active site of the enzyme leading to the involvement of different groups of amino acid residues in the binding and processing of the substrates [17, 18]. Amino acid substitution at position 115 (histidine to tryptophan) and position 192 (glutamine to arginine) in the PON1 gene, and their potential impact on the activity of the enzyme is widely studied [19, 20]. Similarly, two common polymorphic forms of PON2 gene, S311C, and A148G show deficient, normal, intermediate, or increased enzymatic activity [21, 22].

Cholinergic deficiency is closely linked with AD progression [23]. Therefore, various AChEIs play a pivotal role in managing the symptoms and possibly slowing the rate of progression of AD [24]. AChEI reduces the extrasynaptic metabolism of acetylcholine, increases the residence time of the neurotransmitter, and improves postsynaptic stimulation [25]. However, the efficacy of these drugs is hampered as they may cause adverse side effects such as gastrointestinal disturbance, hepatotoxicity, and hypotension [26]. The effectiveness also varies from person to person and is limited in duration [27, 28]. They are not able to completely stop the progression of the disease, and various single-target drugs that have reached clinical trials are not able to effectively treat AD [29, 30]. Therefore, inhibition of AChE remains a promising strategy in AD management, and several studies are being carried out to develop novel AD drugs [31].

Some of the AChEI includes donepezil, rivastigmine, memantine, pyridostigmine, eserine, neostigmine, physostigmine, carbofuran and galantamine [32]. Since these inhibitors play a pivotal role in the treatment of AD, the efficacy of these drugs must be reviewed more often [26]. We have chosen two commonly used inhibitors namely, DHC and PB, and studied the role of PON2 in their efficacy against AD. DHC is a reversible inhibitor of AChE which blocks the hydrolysis of the neurotransmitter Acetylcholine (ACh) and, consequently, increases its activity. This helps in improving neurocognitive function in AD [33]. PB also binds to AChE reversibly and prevents the breakdown of ACh. It also prevents the binding of organophosphates to AChE receptors [34].

A large number of related studies have shown that the main reason for the differences in the clinical efficacy of a therapeutic drug may be closely related to genetic factors [35, 36]. Moreover, though the role of PONs is described as hydrolase enzymes and as antioxidants in several diseases, robust studies clarifying the association between polymorphisms of the PONs gene cluster, including PON2, and enzymatic activities in the neurodegeneration process is still inevident [37, 38]. Here, in this article, a new perspective on the association of the arylesterase activity of the PON2 gene as one of the causes of the ineffectiveness of certain AChEIs against AD is highlighted. Moreover, it provides a concrete analysis of its polymorphic forms whose arylesterase activity may be responsible for the heterogeneous response of individuals to the selected AChEI drugs. This study will help provide an overview of PON2 as a possible pharmacokinetic and pharmacodynamic biomarker of DHC and PB efficacy, and also provide perspectives and limitations within the field of AD therapy.

## Materials and methods

### Primary sequence analysis of HuPON2

Among the paraoxonase family, HuPON1 protein is well studied and properly annotated [39]. Hence, comparative analysis of the primary sequence of HuPON1 and HuPON2 would enable us to know about HuPON2. Both the protein sequences were retrieved from the NCBI protein database. Pair-wise sequence alignment of PON1 and PON2 was performed by using sequence alignment tools of the Discovery studio 4 software. All the conserved residues were identified and considered for further mutational analysis.

### Construction of HuPON2 protein model

Initially, the HuPON2 protein FASTA sequence (Ref Seq ID: NP_000296.2, PDB: 1V04_A) was retrieved from the NCBI protein databank. Consecutively this sequence was utilized to screen the availability of the structural data for HuPON2 in the PDB database. Due to unavailability of the structural data of HuPON2, its model was prepared through a homology modeling approach. Hence, the HuPON2 protein FASTA sequence was used as a query for BLAST analysis. From the PSI-BLAST search list, the crystal structure of Chi-PON1 (G2E6 variant) ranked at the top position, hence it was considered as a template (PDB:1V04) for HuPON2 model preparation. To prepare the template protein, water molecules and hetero atoms were removed from the crystal structure, and all the nonstandard amino acid residues were replaced with corresponding standard residues. Further, this semi-prepared template protein was subjected to energy minimization using the steepest descent algorithm [40]. Discovery Studio software was used to prepare and validate the model quality. Primarily 20 HuPON2 enzyme models were prepared, a further top-ranked model was selected based on the highest negative DOPE score and lowest PDF total energy values. The amino acid residue positions were analyzed through the Ramachandran plot. Finally, the prepared HuPON2 model and the reference 1V04 structures were superimposed, and the C^α^ root mean squared deviation (RMSD) value was recorded to estimate the quality and deviation of the model.

### Selection of AChEI drugs and ligand preparation

**Fig 1:**
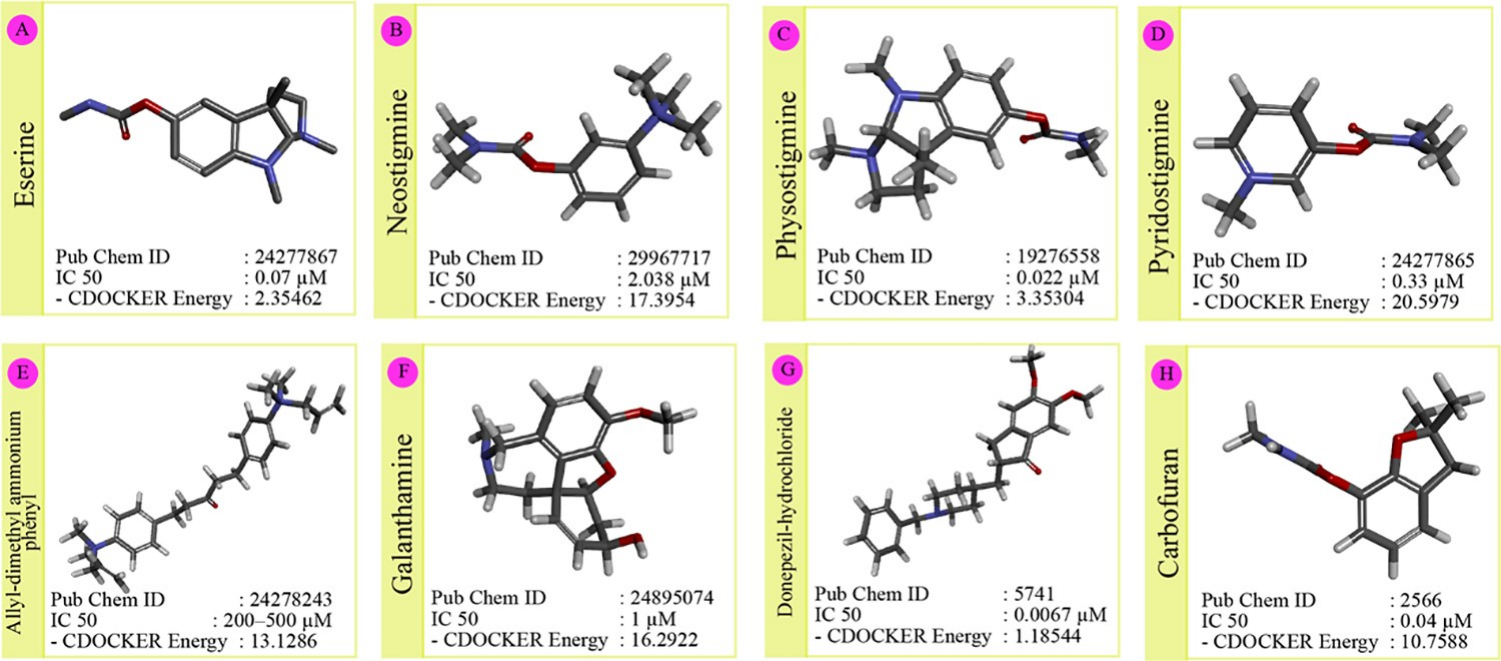
Selection and screening of AChEI drugs. AChEI drugs were screened based on negative CDocker energy and IC_50_ values.

Commercially different drugs are available to treat AD. Eight frequently used AChEI drugs (viz. Eserine, Neostigmine, Physostigmine, Pyridostigmine, Allyldimethylammoniumphenyl, Galanthamine hydrobromide, Donepezil hydrochloride, and Carbofuran) were shortlisted for this study. Unless otherwise available in the PubChem webserver, all drugs were modeled using ChemSketch software. All the drugs were subjected to energy minimization followed by the assignment of proper charges to bring them into an active state to carry out the docking study. Energy minimization and charge assignment were performed by Discovery studio 4 software. Out of the eight inhibitors, two drugs based on negative CDOCKER energy and IC50 values available from the literature were finally screened (Fig 1).

### Docking with AChEI drugs

Molecular docking studies were performed using the Discovery Studio software suite. Each of the dockings was performed with different drug molecules but in the same default parameters (VDW = 4.0 and H-bonding = 2.5 Å). CDOCKER algorithm of the Discovery studio software was used to execute the experiment, which is a grid-based highly accurate molecular docking method that employs CHARMm [41]. All the binding site residue coordinates from the interface of HuPON1 ligand complex (PDB:1V04) structures were retrieved. This coordinate value enables the recognition of the corresponding residues and their local clusters at HuPON2, which precisely locate the input site sphere. It is a pipeline pilot, define the sphere as x, y, z, and r where x, y, z specify the coordinates of the centre, and r is the radius of the sphere. It allows docking drug compounds at the targeted site i.e. active site. After completion of docking, all the docked poses for each of the drugs were ranked based on the CDOCKER energy score. Higher the negative CDOCKER energy score, the more the favourable binding, and this function arrange all the competent poses in certain clusters. The maximum number of poses and/or lowest C^α^ RMSD values were used to sort out the best pose.

### Cloning of HuPON2 gene

HuPON2 gene (NM_000305.3) was synthesized from Sino Biological Inc. The synthetic HuPON2 gene was PCR amplified with a pair of primers (S1 Table) that were flanked by two different sets of restriction sites. The PCR product was then cloned in the pJET1.2 PCR cloning vector, and digested with the specific set of restriction enzymes to prepare targeted HuPON2 inserts having *NheI/XhoI* restriction sites for subcloning. The resulting HuPON2 inserts were then eluted from the agarose gel, purified using MinElute gel extraction kit (Qiagen), and finally sub-cloned into the pET28a(+) vector under the control of T7 promoter using *Nhe*I and *Xho*I restriction sites (S1 Fig). Similarly, vector pET28a was prepared by digesting with the same restriction enzymes. The ligation was then performed using T4 DNA ligase, and the ligation mixture was subsequently transformed into *E*. *coli* (XL-1 blue). The colonies were checked for positive clones using colony PCR and further by restriction digestion. Final confirmation of the positive clone was done by Sanger DNA sequencing.

### *In vitro* HuPON2 mutant creation and screening of positive mutants

Starting from the HuPON2+pET28(a) plasmid construct, and based on the sequence alignment analysis, two sets of mutants were created. The first consisted of two catalytic site mutants, His 115 and Lys 192 which were mutagenized into Trp and Glu respectively. The other set consisted of two polymorphic site mutants, Ala 148 and Ser 311 which were mutated to Gly and Cys respectively. For these site-directed mutageneses, the Platinum® *Taq* DNA Polymerase High Fidelity (Invitrogen) and mutagenic primers for overlapping PCR were used. The template DNA was removed from the PCR product by *Dpn*I (New England Biolab) digestion following the manufacturer’s instructions. The *DpnI* digested PCR products were transformed into *E*. *coli* XL-10 competent cells. The mutant plasmids designated as HuPON2-H115W, HuPON2-K192Q, HuPON2-A148G, and HuPON2-S311C were verified by Sanger sequencing to check that only the desired mutations were introduced during the amplification procedure. The complementary pairs of oligonucleotides used in this study for subcloning and site-directed mutagenesis are listed in the S1 Table.

### Expression and extraction of HuPON2 WT and mutant proteins

*Escherichia coli* Rosetta (DE3) cells were transformed with the WT and mutant plasmids, namely HuPON2-WT, HuPON2-H115W, HuPON2-K192Q, HuPON2-A148G, and HuPON2-S311C. The transformed cells were grown overnight in LB agar plates in the presence of 50 μg/mL kanamycin for 14–16 hours at 37°C. A single colony from each plate was then inoculated in three different LB broths with 50 μg/mL of kanamycin. The primary cultures grown overnight were re-inoculated in fresh LB broth with the corresponding antibiotic, and followed to grow at 37°C till OD_600_ was 0.4–0.6. All of the secondary cultures were grown at 37°C were then induced with IPTG 1.0 mM concentration, and grown at 37°C for 6 hours. The cells were then harvested by centrifugation at 4000*g*, and the cell pellets were re-suspended with lysis buffer containing 50 mM Tris–HCl pH 8.0, 1 mM CaCl_2_, 0.5% Triton X-100, 1.0 mg/mL lysozyme, and 1 mM PMSF. Sonication was then performed on cell lysates at 30% amplitude for the 20 seconds, 5–6 times with 20 seconds intervals to obtain a clear solution. It was then centrifuged at 12,000*g*, supernatant and pellet were collected separately. An equal volume of lysis buffer (without lysozyme) was used to re-suspend the pellet. Finally, the expression level of HuPON2 in all supernatants and pellets was checked on SDS-PAGE.

### Purification of HuPON2 protein through inclusion body (IB) solubilization

Since most of the proteins were in the pellet portion, proteins from inclusion bodies (IBs) were purified using a mild solubilizing agent guanidinium hydrochloride (GdnHCl) [42]. For each 1 L culture cell pellet, 130 mL resuspension buffer containing 50 mM Tris-HCl pH 8.0, and 1 mM PMSF was used. 4.0 mL lysozyme (10 mg/mL) was then added into the resuspended cells and kept at room temperature for 1 hour on a rocker. Subsequently, 20 mL of 5M NaCl was added to it and shaken well. Then 10 mL of 25% Triton X-100 was added, mixed well, and then incubated at room temperature on a rocker for 30 minutes, shaking periodically. The solutions were then sonicated at 30% amplitude 20-seconds on/off cycle for six times followed by centrifugation at 10,000 rpm for 20 minutes at 4˚C. The supernatants were stored as washes, and the pellets were resuspended in 100 mL buffer containing 50 mM Tris-HCl pH 8.0, 1 mM PMSF, and 2 mL of 25% Triton X-100. This was repeated four times with the same buffer without triton. Finally, IB solubilization buffer containing 4M GdnHCl, 100 mM Tris pH 8.1, and 10 mM DTT at greater than 5 mg/mL approximate protein concentration (measured by BCA protein assay kit) was added to the pellet. It was then incubated for 2 hours on the rocker with periodic shaking. Centrifugation at 10,000 rpm for 20 minutes at 4˚C was done and the supernatant and pellet (if any) were collected separately. The concentration of the proteins was taken using NanoDrop (ThermoScientific, USA) using extinction co-efficient for Abs 0.1% (= 1 g/L) as 0.809 (ProtParam tool, Expasy) and assuming all Cys residues to be reduced.

### UV-Visible spectroscopy for protein folding

Various methods have been developed to extract protein structural information from UV-Visible spectra [43]. Spectral changes were plotted based on binding of varied concentrations of a divalent cation, Calcium ion as cofactor using UV-Visible titration following the method described earlier [44]. HuPON2-WT IB purified proteins were incubated with different concentrations of Calcium ions. Protein samples were prepared in buffer (50 mM Tris-HCl pH 8.0, 0.1% Triton X-100) with varied metal concentrations (50 μM, 100 μM, and 500 μM). The background was measured by adding 1mM EDTA to chelate all the metals from the protein sample. The prepared samples were incubated for 20 minutes and then a UV-Visible spectrophotometer (Lambda 45 from Perkin Elmer) was used to perform the scanning of the protein metal complex ranging from 280 nm to 380 nm wavelength. Then, delta absorbance was calculated by subtracting the background level of absorbance.

### Enzyme activity with Phenylacetate and γ-Thiobutyrolactone

Proteins purified from IBs were used to perform arylesterase and lactonase activities, using Phenylacetate and γ-Thiobutyrolactone (GTBL) (Sigma Aldrich) as substrates respectively. Enzyme activity was performed in activity buffer containing 50 mM Tris-HCl pH 8.0 and 1 mM CaCl_2_. All the reactions were performed in triplicates. The reactions were set up in 96 well plates keeping the overall reaction volume 200 μL.0.1 mM 5,5’-Dithiobis, 2-nitrobenzoic acid (DTNB) was used as an indicator for lactonase activity. 0.5 mM of phenylacetate and 1mM of GTBL was used as the final concentration. The substrate dilutions were prepared in activity buffer by keeping the final volume as 100 μL for each reaction and indicator stock was prepared in DMSO. Enzymes were also diluted in activity buffer separately to keep the volume 100 μL and were added to substrate just before taking the readings. The absorbance was taken at 320 nm for Phenylacetate and 412 nm for GTBL with intervals of 1 minute for 30 minutes using multimode micro-plate Reader (BioTek Synergy HT). The product formation in 30 minutes was plotted using the respective absorbance values.

### Enzyme kinetics of WT and mutants with Phenylacetate

Enzyme kinetics were performed following the modified methods reported by Kondo et al. [45, 46]. IB purified proteins were used to perform arylesterase activity, for which, Phenylacetate (Sigma Aldrich) was used as a substrate. Enzyme kinetics was performed in activity buffer containing 50 mM Tris-HCl pH 8.0; 1 mM divalent metal, Calcium. Different substrate concentrations (2.5 mM, 2 mM, 1.5 mM, 1 mM, 0.5 mM, and 0.1 mM) of each were used in the reactions, and equal concentration of proteins, WT, and mutants, were used in the reaction. All the reactions were performed in triplicates in 96 well plates keeping reaction volume 200 μL. The substrate was prepared in activity buffer, keeping the volume 100 μL for each reaction. Enzymes were also prepared in activity buffer separately in 100 μL and were mixed with substrate just before taking the readings. The kinetics was performed at 320 nm up to 60 minutes with intervals of 1 minute using a multimode microplate reader (BioTek Synergy HT). The product formation (OD/min) was plotted using a double reciprocal graph (Lineweaver Burk plot) and Vmax & Km values were estimated. The graph was plotted to take the average values of each triplicate with the standard error of the mean (SEM) in the plot. Further, Vmax & Km values were converted to μM/min and μM respectively taking extinction coefficient value as 176 M^-1^ cm^-1^.

### AChE assay to detect hydrolysis of Donepezil hydrochloride and Pyridostigmine bromide by HuPON2 and its mutants

HuPON2 and mutants hydrolyzing the inhibitors, DHC and PB, were quantified using Amplex Red Acetylcholine/Acetylcholinesterase Assay Kit (Invitrogen) following the manufacturer’s instructions. IB purified WT and mutant HuPON2 (10 μL each expressed and purified under the same conditions) were preincubated with 2.5 μM DHC and PB (Sigma Chemical St Louis, MO) separately, for 10 minutes at room temperature. AChE activities were then measured using Amplex Red Acetylcholine/Acetylcholinesterase Assay Kit (Invitrogen) in the presence and absence of HuPON2. The sample without HuPON2 was used as a control. The absorbance was taken at 571 nm to measure the activity.

### AChE kinetics to detect AChE inhibition by Donepezil hydrochloride and Pyridostigmine bromide in the presence of HuPON2-WT

AChE kinetics were performed following the Amplex Red Acetylcholine/Acetylcholinesterase Assay Kit (Invitrogen) manufacturer’s instructions with modifications. Different substrate (ACh) concentrations (0 μM, 20 μM, 40 μM, 60 μM, 80 μM, and 100 μM) were used to perform kinetics. Experiments were performed in three sets: The first set was without AChEI and HuPON2, the second set was with AChEI only and the third set was with both AChEI and HuPON2-WT. All the reactions were performed in triplicates in 96 well plates keeping reaction volume 200 μL. The kinetics was performed at 571 nm up to 60 minutes with intervals of 1 minute using a multimode microplate reader (BioTek Synergy HT). The product formation (OD/min) was plotted using a double reciprocal graph (Lineweaver Burk plot) and Vmax & Km values were estimated. The graph was plotted to take the average values of each triplicate with the standard error of the mean (SEM) in the plot. Further, Vmax & Km values were converted to μM/min and μM respectively taking extinction coefficient value as 58 mM^-1^ cm^-1^ [47].

### Statistical analysis

GraphPad Prism version five statistical program (GraphPad Software, San Diego, CA, USA) was used to statistically analyze the data obtained. Results are represented as mean±SE. ANOVA (Tukey post hoc test for comparing 3 groups), t-test (for comparing 2 groups), chi-square (for non-parametric), and Pearson correlation were adopted to evaluate differences among examined samples. Statistical significance was set at p < 0.05.

## Results

### HuPON2 model structure

**Fig 2:**
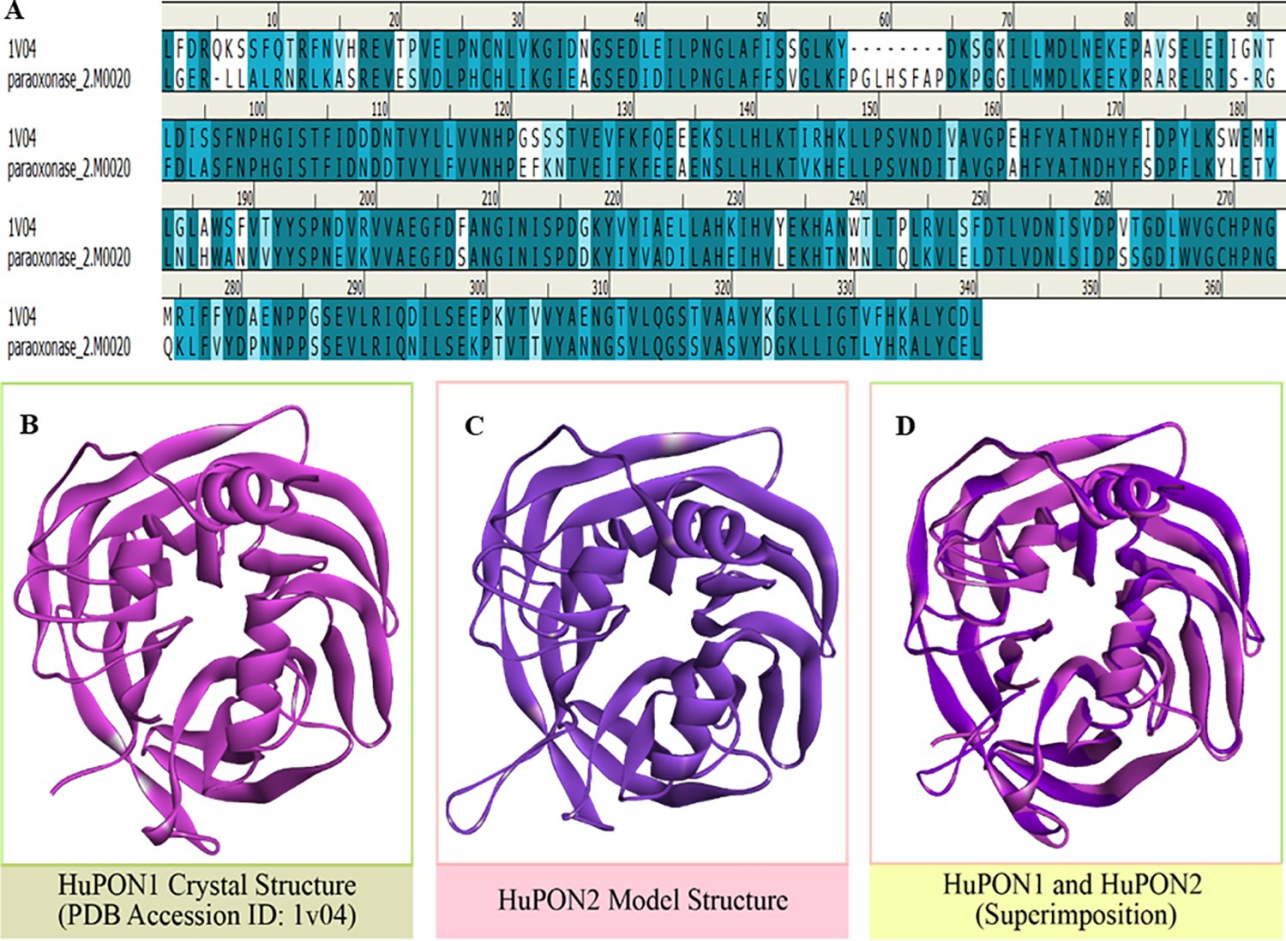
*In silico* analysis of HuPON2. A. Sequence alignment of HuPON2 on Chi-PON1 (PDB:1V04) shows 64.7% sequence identity and 82.6% sequence similarity. B. Crystal structure of HuPON1 (PDB:1V04); magenta color-coded. C. Homology model of HuPON2, prepared from HuPON1 template (PDB:1V04); violet color-coded. D. Superimposition of HuPON1 (magenta) and HuPON2 (violet), shows the close similarity of both the structures, RMSD value of 0.543. The analysis was performed in Discovery Studio 4.0.

The model structure of HuPON2 was prepared from the Chi-PON1 (PDB:1V04) crystal structure. Initially, the target protein sequence was aligned with the template. HuPON2 protein sequence showed 64.7% sequence identity and 82.6% sequence similarity with HuPON1 (PDB:1V04) (Fig 2A). Ultimately twenty structural models were prepared from the target protein sequence and ranked based on the PDF total energy, PDF physical energy, and DOPE score. The model having the lowest PDF total energy and highest -DOPE score was selected for further evaluation. In this aspect, the 20^th^ number model fulfilled the criteria and ranked top of the rest of the models (PDF Total Energy = 2154.3918, PDF Physical Energy = 1102.9973, and DOPE Score = − 40602.5390) (S2 Table). A predictive analytical platform was used to identify the location of different secondary structure formations in the model structure (S2A Fig). Loop refinement was performed to minimize the C^α^ RMSD and the target models were evaluated through superimposition and Ramachandran plot (S2B Fig). Upon superimposition of the HuPON2 model structure with HuPON1 (as a reference) crystal structure enables to quantify the main chain atoms RMSD value of 0.543 (Fig 2D). All this parametric analysis and observation validates the model and increases confidence to obtain correct results.

### Docking and filtration of the docked poses

**Fig 3:**
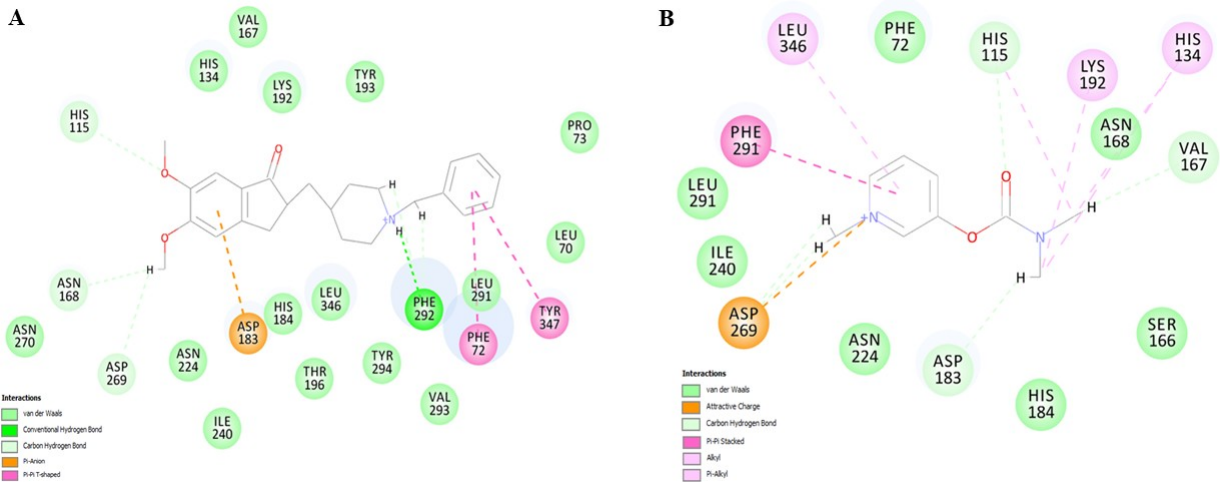
Docking of drug compounds in HuPON2. A. *In silico* docking of the 3D structure of HuPON2 with DHC and B. *In silico* docking of the 3D structure of HuPON2 with PB. Various interacting residues are shown and the type of their interaction is color-coded. Discovery studio software was used for the visualization of the results and the creation of images.

CDOCKER program of the Discovery Studio software was used to dock the drug compounds on the HuPON2 model. More than a thousand docked poses were obtained, top-ranked 25 poses were analyzed. *In situ* ligand minimization algorithm was used to estimate the energy requirement at the local position. The yellow surface ball indicates the location of the active site pocket in the HuPON2. The coordinates of the surface ball are X = − 7.009000, Y = − 20.241000, Z = 32.861000, and R = 8.614570 Å (S3 Fig). In the catalytic site, all the active residues were functioning in a cluster. Docking study demonstrates that different drug compound occupies a varied space in the active location hence their binding efficiency differs from each other so as the CDOCKER Energy (S4 Fig and S3 Table). The docking analysis revealed the involvement of His 115; His 133 catalytic dyad interacting residues, and also includes the mutational site K192 under study. Together, these observations suggested that HuPON2-WT, H115 and K192 are important in determining substrate binding and specificity, and are likely to be involved in substrate hydrolysis (Fig 3).

### HuPON2-WT clone

The HuPON2 insert gene was PCR amplified with the specific restriction site primers and cloned into the pJET1.2 cloning vector. The recombinant plasmid was further subcloned in the pET28a(+) expression vector. The resulting recombinant plasmid, i.e., HuPON2-pET28a(+) (S1 Fig) was subjected to restriction digestion with the corresponding set of restriction enzymes to confirm the proper ligation. In agarose gel electrophoresis, the recombinant showed fall out at around 1000 bp DNA ladder that confirmed proper integration of the desired HuPON2 insert of 1065 bp length in the target expression vector (S5 Fig).

### HuPON2 mutants

To investigate the putative effect of the substitutions at H115 and K192 positions, the aforementioned mutations were introduced in the background of a full-length WT HuPON2 clone via site-directed mutagenesis. Overlapping primers with the introduced mutation was used for PCR amplification (S1 Table). The PCR amplified product was digested with *DpnI* to digest the WT plasmid. To check for the complete digestion of the template plasmid, the *DpnI* digested PCR products were electrophoresed on agarose gel (S6A Fig). No band was found for the template plasmid as seen in the control. This was further confirmed by no colonies on the transformed control plates. Subsequent sequencing of the mutated plasmids confirmed the presence of the introduced mutations. Polymorphic site mutants, A148G and S311C, were also created in the same manner (S6B Fig).

### Expression and purification of HuPON2 WT and mutant proteins

To check if the constructed clones were functional, *E*. *coli* rosetta cells were transformed with the plasmid of WT and mutant HuPON2 (HuPON2-WT, HuPON2-H115W, HuPON2-K192Q, HuPON2-A148G, and HuPON2-S311C). HuPON2 proteins, both WT, and mutants were expressed in *E*. *coli* rosetta cells in LB growth media at different temperatures. None of the conditions had HuPON2 expressed in soluble form. Unfortunately, the HuPON2 expression in the insoluble fraction was remaining almost unchanged as it was in *E*. *coli* under normal expression conditions. Hence, mild-solubilization and denaturation of HuPON2 IBs were done (S7 Fig). To study the folding of IB purified protein, UV-Visible spectroscopy was done for HuPON2-WT protein in the presence of a varied concentration of Calcium ion. HuPON2-WT protein with EDTA was used as a control. A concentration-dependent shift in the absorbance was observed at around 340 nm (S8 Fig).

### HuPON2 and the efficacy of selected AChEI drugs

**Fig 4:**
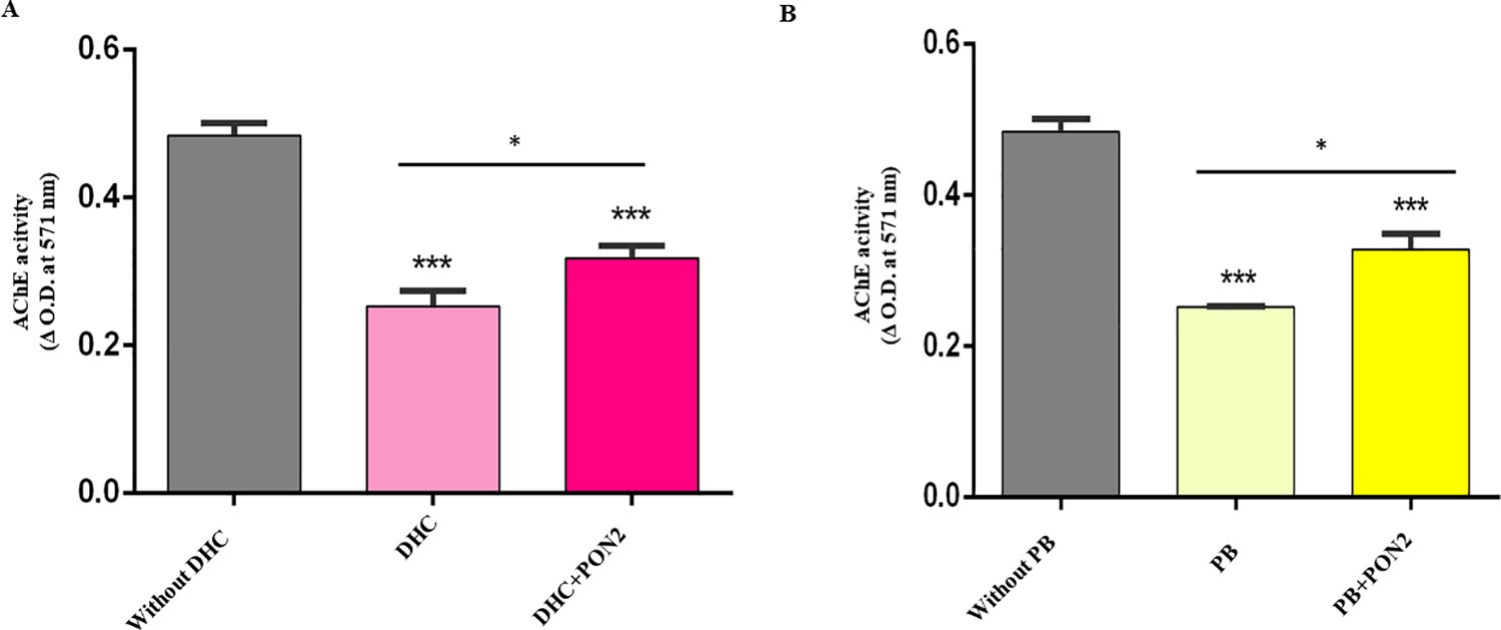
Comparison of AChE activity in the presence of inhibitors and enzymes. A. DHC, in the presence and absence of HuPON2, B. PB, in the presence and absence of HuPON2. Inhibition is shown for AChE activity in the absence of inhibitors and HuPON2.

The amount of substrates, DHC and PB, inhibited by the enzyme was quantified by the amount of AChE activity. AChE activities were measured using Amplex Red Acetylcholine/Acetylcholinesterase Assay Kit (Invitrogen) in the absence of both inhibitor and HuPON2, in the presence of only the inhibitor, and in the presence of both inhibitor and HuPON2. The greater the AChE activity, the greater is the degradation of the substrate by the enzyme and vice versa. It was observed that HuPON2 affected the efficacy of selected AChEI drugs (Fig 4).

### Esterase and lactonase activities of WT and catalytic mutants of HuPON2

**Fig 5:**
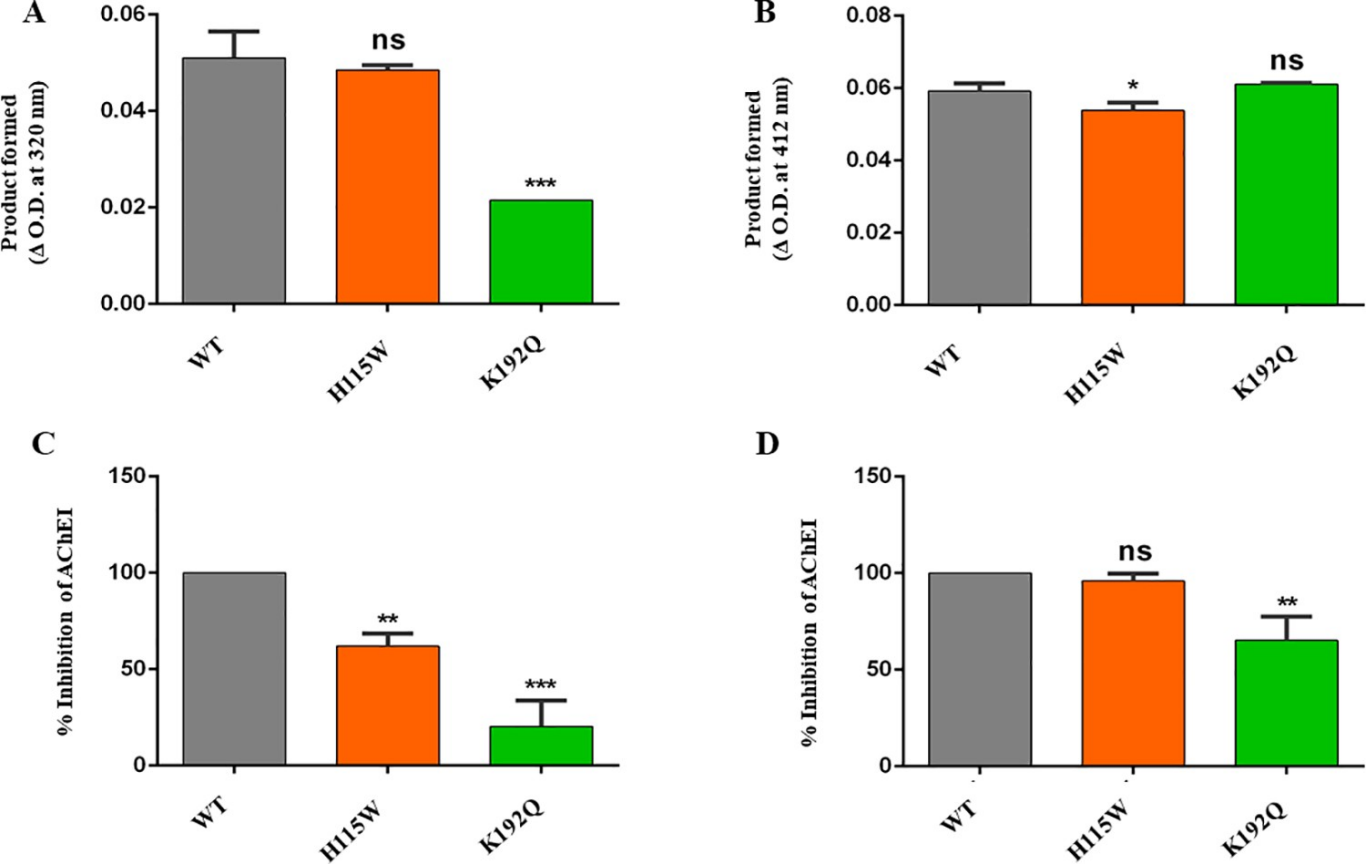
Comparative activities of HuPON2-WT and catalytic site mutants and corresponding inhibition of selected AChEIs. A. Esterase activity of HuPON2-WT compared with catalytic site mutants, B. Lactonase activity of HuPON2-WT compared with catalytic site mutants. The catalytic activity was followed in intervals ranging from 0 to 30 minutes. The activities were reported as product formed in terms of initial and final absorbance measured. Inhibition of AChEI by C. DHC and D. PB respectively in the presence of purified HuPON2-WT and mutant proteins. The well without HuPON2 protein was used as a control. Inhibition was reported as a percentage to control wells read. Results are the average of triplicates and error bars show the standard deviation.

To compare the functional characteristics of the expressed WT and mutant PON2, the catalytic activity using Phenylacetate for arylesterase activity and GTBL for lactonase activity as substrates were analysed. A decrease in esterase activity in the case of catalytic mutants was observed. Although there was no significant decrease in the esterase activity of H115W mutant, a significant decrease in the case of the K192Q mutant was observed. In the case of lactonase activity, a significant decrease in activity in the H115W mutant, and a not-so-significant increase in activity in the K192Q mutant were observed. Therefore, it can be inferred that H115W is responsible for the lactonase activity and K192Q for the esterase activity of the enzyme (Fig 5A and 5B).

### Hydrolysis of Donepezil hydrochloride and Pyridostigmine bromide by WT and catalytic mutants of HuPON2

As shown above, H115W and K192Q mutations affected the esterase activity of the enzyme, a corresponding decrease in inhibition of selected inhibitors by these mutants were observed (Fig 5C and 5D). Hydrolysis of DHC and PB by WT and catalytic mutants was quantified as the percentage of inhibition of AChEI, considering AChEI inhibition in the presence of HuPON2-WT to be 100%. The more the AChEI inhibition, the lesser is the efficiency of inhibitors, and the more will be AChE activity i.e. more inhibitors are hydrolyzed by the enzyme. In the case of DHC, the decrease in AChEI inhibition was highly significant for both the mutants, however in the case of PB, the decrease was more significant for the K192Q mutant. Therefore, the mutants could not hydrolyse the inhibitors as efficiently as WT and so AChE activity is inhibited. In particular, H115W showed about 62% AChEI inhibition, and K192Q about 20%, WT being 100% in the case of DHC; whereas in the case of PB, H115W showed about 96%, and K192Q about 65% AChEI inhibition, WT being 100%. This has proven that the K192Q mutation decreased the esterase activity, and thus the enzyme became less efficient in inhibiting the drugs. As a result, AChEI inhibition is decreased. Thus, it can be inferred that HuPON2 might affect the effectiveness of these inhibitors through its esterase activity.

### Esterase and lactonase activities of WT and polymorphic HuPON2

**Fig 6:**
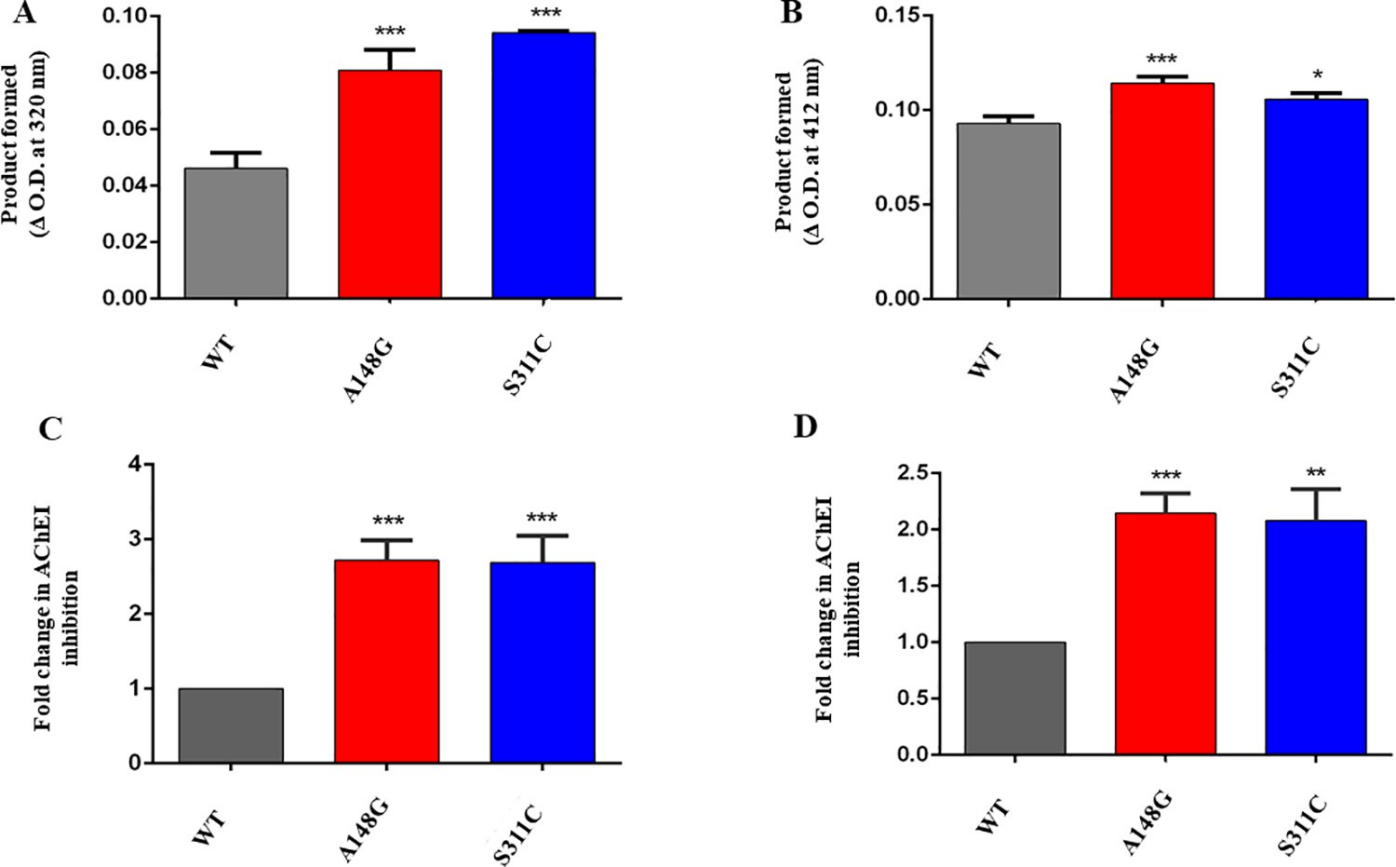
Comparative activities of HuPON2-WT and polymorphic site mutants and corresponding inhibition of selected AChEIs. A. Esterase activity of HuPON2-WT compared with polymorphic site mutants, B. Lactonase activity of HuPON2-WT compared with polymorphic site mutants. The catalytic activity was followed in intervals ranging from 0 to 30 minutes. The activities were reported as product formed in terms of initial and final absorbance measured. Inhibition of AChEI by polymorphic HuPON2. C. DHC and D. PB in the presence of purified HuPON2-WT and polymorphic site mutant proteins. The wells without HuPON2 protein were used as control. AChEI drug inhibition was reported as a fold change to WT. Results are the average of triplicates and error bars show the standard deviation.

To compare the functional characteristics of the WT and polymorphic HuPON2, the catalytic activity of the expressed polymorphic proteins was analysed using Phenylacetate and GTBL as substrates for arylesterase and lactonase activity respectively. A significant increase in both arylesterase and lactonase activity of mutants was observed. Between A148G and S311C, the latter showed greater arylesterase activity. However, there was not much difference in lactonase activity between the two mutants (Fig 6A and 6B). It can be inferred that both the polymorphisms, 148G and 311C, have greater arylesterase and lactonase activity.

### Hydrolysis of Donepezil hydrochloride and Pyridostigmine bromide by WT and polymorphic mutants of HuPON2

To further check whether HuPON2 polymorphism is associated with the efficiency of AChEI drugs against AD, an AChE assay with the purified polymorphic proteins was performed in the same way as was for catalytic site mutant proteins. Interestingly, there was a more than 2.5 fold increase in inhibition of DHC by both the mutants. In the case of PB, A148G inhibited the drug by 2.2 fold, and S311C by 2 fold as compared to WT. In both the cases of drugs, the increase in inhibition was highly significant for both the mutants, but in the case of PB, the increase was lesser as compared to that in DHC (Fig 6C and 6D). Thus, HuPON2 polymorphism may be responsible for “responders” and “non-responders” to AChEI therapy. Gly at 148 and Cys at 311 positions increased the esterase activity, and thus the enzyme became more efficient in inhibiting the drug in these cases. Both the mutants showed almost a similar increase in esterase activity. However, the trend in lactonase activity did not seem to correspond with the trend in AChEI inhibition. Both the drugs are almost equally inhibited by the mutants.

### Enzyme kinetics to detect AChE inhibition by Donepezil hydrochloride and Pyridostigmine bromide in the presence of HuPON2-WT

AChE enzyme kinetics was performed with the HuPON2-WT protein. Vmax and Km were calculated respectively and summarized in Table 1. Since arylesterase activity of HuPON2 is responsible for the hydrolysis of AChEIs, enzyme kinetics was performed for HuPON2-WT and all four mutants with Phenylacetate. Vmax and Km values for the WT and mutant PON2 are summarized in Table 2. Vmax of AChE in the absence of both AChEI and HuPON2 was the highest, whereas that with only AChEI was the lowest for both the inhibitors. Km values were also found to be higher in the presence of inhibitors only suggesting decreased specificity. However, Km values are lower for AChE with both AChEI and HuPON2 as compared to that with only inhibitors for both the AChEIs suggesting higher specificity. Though, Vmax is lower in the presence of both HuPON2 and either of the inhibitors as compared to that in the absence of both HuPON2 and inhibitors.

**Table 1:** AChE enzyme kinetics.

AChEIs	Kinetic parameters	ACh	ACh+AChEI	ACh+AChEI+PON2
**DHC**	Vmax (μM/min)	3.51±0.48	0.47±0.009	1.06±0.29
** **	Km (mM)	14.83±2.11	110.49±5.64	9.35±2.56
**PB**	Vmax (μM/min)	3.51±0.48	0.28±0.002	0.48±0.03
** **	Km (mM)	14.83±2.11	52.73±2.46	42.93±3.45

Double reciprocal plot was used for the calculation of kinetic parameters (Km and Vmax).

**Table 2:** Enzyme kinetics for WT and mutant HuPON2 with Phenylacetate as substrate.

Proteins	Vmax (μM/min)	Km (μM)
**WT**	6.22±0.34	10.19±0.09
**H115W**	6.49±0.09	236.89±25.39
**K192Q**	3.12±0.05	13.60±1.51
**A148G**	7.95±0.64	13.24±11.34
**S311C**	12.38±1.29	176.96±49.71

Double reciprocal plot was used for the calculation of kinetic parameters (Km and Vmax).

Similarly, the kinetics data for the HuPON2-WT and mutants were observed to be in the same order as their activity was obtained. Vmax for K192Q is significantly lower than the WT whereas that of H115W has no significant change. Both the polymorphic site mutants have higher Vmax values as compared with the WT. However, Km values obtained were varied with WT having the lowest Km suggesting its highest affinity and H115W mutant with highest Km suggesting lowest affinity.

## Discussion

AChEIs are the preferred treatment for mild or moderate AD, but only a subgroup of patients taking these inhibitors attain clinically relevant improvement [48]. Several factors may modify the response to treatment, gene polymorphism is one among them. Genetic factors may account for an estimated 60–90% of the variability in the disposition and pharmacodynamics of AChEIs [49]. Identifying an unambiguous marker in patients responding to cholinergic therapy would be a valuable finding.

Studies investigating the association of PON2 activity with diseases have been scarce because of the unknown true biological substrate and lack of well-established methods [12]. Therefore, an indirect quantitative assay to quantitate the amount of substrate, DHC, and PB, hydrolyzed by the HuPON2 enzyme was used in this study. AChE activity or ACh was detected in a fluorescence microplate reader in an ultrasensitive manner using the Amplex® Red Acetylcholine/Acetylcholinesterase Assay Kit (Sigma A12217) [50]. A HuPON2 homology model was created on the PON1 template, selected AChEI drugs were docked with the modeled PON2 and interacting residues were analyzed based on docking results. This helped in the context of the development of mutants for proving the proposed hypothesis and permits postulation of the catalytic activity responsible for the hydrolysis of selected AChEIs.

Moreover, the association of the PON2 gene in the responsiveness against AChEI therapy in AD was focussed on. Klimkowicz *et*. *al*. previously demonstrated that PON1 gene polymorphisms do not influence the response to treatment in AD [51]. So far, no study has investigated the relationship between responsiveness to the PON2 gene and cholinergic therapy. Herein, the role of the HuPON2 gene in responsiveness against cholinergic therapy is shown for the first time. This nature of PON2 could be due to its esterase activity.

In support of the postulated hypothesis, the catalytic sites H115 and K192 were investigated based on bioinformatic analysis of HuPON2 with extensively studied PON1 [15]. H115 is a major active site residue and Q192R polymorphism of PON1 has been shown to affect AChEI therapy [52, 53]. The mutagenesis data indicated that both arylesterase and lactonase activities of HuPON2 were catalyzed by H115 and K192 residues. Both the mutations reflect in the metabolism of screened AChEI drugs. Though the H115W mutant showed a milder decrease, K192Q mutation resulted in a highly significant, decrease in esterase activity. It was found that the catalytic site mutants affect the esterase activity of the enzyme and thus metabolize the AChEIs (DHC and PB) but less efficiently than WT. This shows that the esterase activity of HuPON2 might be responsible for AChEI drug inhibition.

Further, two common PON2 polymorphisms were analyzed similarly. The polymorphic forms, 311C, and 148G showed higher esterase activity than the WT and thus inhibited the function of AChEIs in the same manner. Therefore, the HuPON2 gene might influence the responsiveness to these drugs mainly through its esterase activity. This was also validated with the help of polymorphic site mutants. Hence, HuPON2 could prove to be a prognostic indicator of individual response to treatment in AD patients.

In summary, the present study revealed HuPON2 as a novel enzyme affecting the efficacy of AChEIs, DHC, and PB, through its esterase activity. Catalytic site mutants of the enzyme that affected its esterase activity also affect its AChEIs metabolizing efficiency. Furthermore, the polymorphic forms of PON2, A148G, and S311C were shown to have increased esterase activity, and thus proven to be more efficient in making the AChEIs ineffective against the disease. Thus, HuPON2 could be a major prognostic indicator for “responders” or “non-responders” to DHC and PB therapy.

## Supporting information

S1 Fig Complete map of HuPON2-pET28a(+) plasmid. The HuPON2 gene was cloned using *Nhe*I and Xh*o*I restriction sites of pET28a(+). The complete recombined map was created with SnapGene.
(TIF)

S2 Fig Primary analysis of the HuPON2 sequence and the model. A. Predictive secondary structural analysis of the HuPON2 protein sequence. Orange and blue color beneath the primary sequence symbolizes the probability of alpha-helix and beta-sheet occurrence respectively. B. HuPON2 model was analyzed through Ramachandran plot. None of the residues were found in the disallowed region.
(TIF)

S3 Fig Docking of drug compounds in HuPON2. A. HuPON2 model protein was prepared for docking purpose, B. Catalytic site identification by yellow sphere ball. Green dots represent all the possible sites for drug docking, C. Best 10 docked poses of Eserine drug were filtered and represented in this panel. The docked pose is the first among the 10 times docking with different conformations of the protein. Discovery studio software was used for the visualization of the results and the creation of images.
(TIF)

S4 Fig CDOCKER energy for 10 best docking conformations of the docked ligand molecules. A. The docking library of all the screened drugs with HuPON2. B. The topmost stable conformations considered from the docking library were obtained.
(TIF)

S5 Fig Cloning of HuPON2 into vector pET28 a (+). Agarose gel images showing, A. The PCR amplified product of HuPON2 gene, band size 1065 bp, gradient PCR was set at different temperatures (60֯C, 64֯C, 68֯C, 70֯C) B. confirmation of HuPON2 gene cloning in pJET1.2 vector by restriction digestion, C. confirmation of HuPON2 gene cloning in pET28a(+) vector by restriction digestion.
(TIF)

S6 Fig Creation of HuPON2 mutants. The efficiency of Dpn1 digestion of the PCR products is shown. The PCR products of plasmid pET 28(a)-HuPON2-WT with the indicated mutations, A. H115W, K192Q, and WT primer PCR control (CNTRL), B. A148G, S311C, and WT primer PCR control (CNTRL) were left untreated (-) or treated (+) with restriction enzyme Dpn1 and then analyzed by 1% agarose gel electrophoresis. Complete digestion of the WT plasmid can be seen in the control.
(TIF)

S7 Fig Band pattern of HuPON2 proteins on SDS-PAGE. A. Expression at different temperatures showing most of the fractions in pellet, B. The soluble HuPON2 WT achieved by mild solubilization and denaturation, C. catalytic site mutant proteins solubilized in the same manner as WT and D. polymorphic site mutant proteins solubilized in the same manner as WT.
(TIF)

S8 Fig UV-Visible titration for HuPON2-WT. Different concentrations of Ca^2+^ ions were taken. UV-Visible data were acquired at 280–380 nm wavelengths. A concentration-dependent shift in the delta absorbance was observed at around 340 nm.
(TIF)

S1 Table List of the primers used. (DOCX)

S2 Table Ranking of all the prepared models of HuPON2. The ranking is based on PDF total energy, PDF physical energy, and DOPE score. Among all these prepared models, the 20^th^ number model is the best one.
(DOCX)

S3 Table CDOCKER energy of the topmost stable docked conformations. (DOCX)
