# Supplementary figures and images for: Association between human paraoxonase 2 protein and efficacy of acetylcholinesterase inhibiting drugs used against Alzheimer’s disease
Source: PLoS One. 2021 Oct 29;16(10):e0258879. doi: 10.1371/journal.pone.0258879 (PMC8555796; doi:10.1371/journal.pone.0258879)

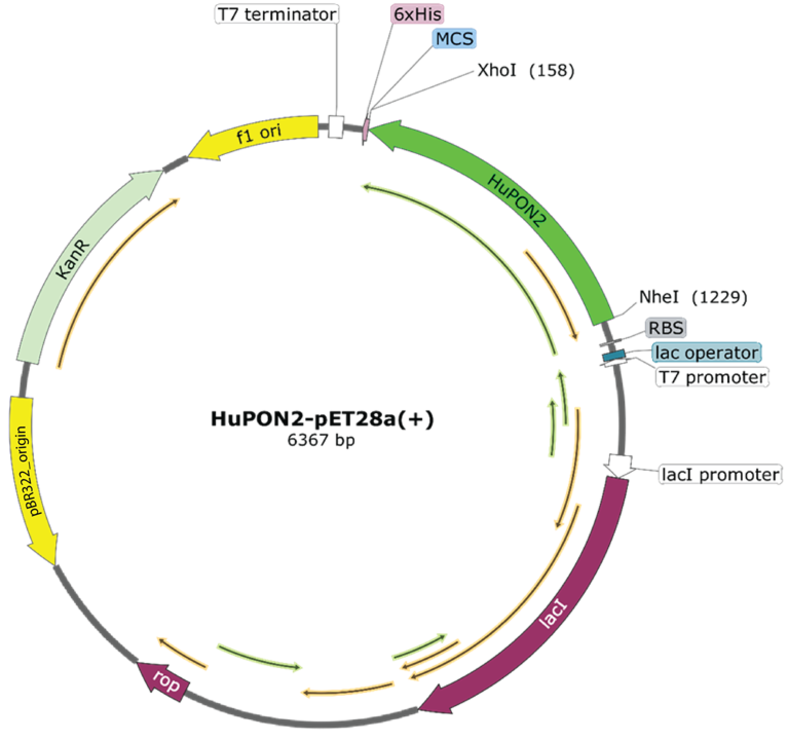

Supplement: S1 Fig — The HuPON2 gene was cloned using NheI and XhoI restriction sites of pET28a(+). The complete recombined map was created with SnapGene. (TIF) [file pone.0258879.s001.tif]

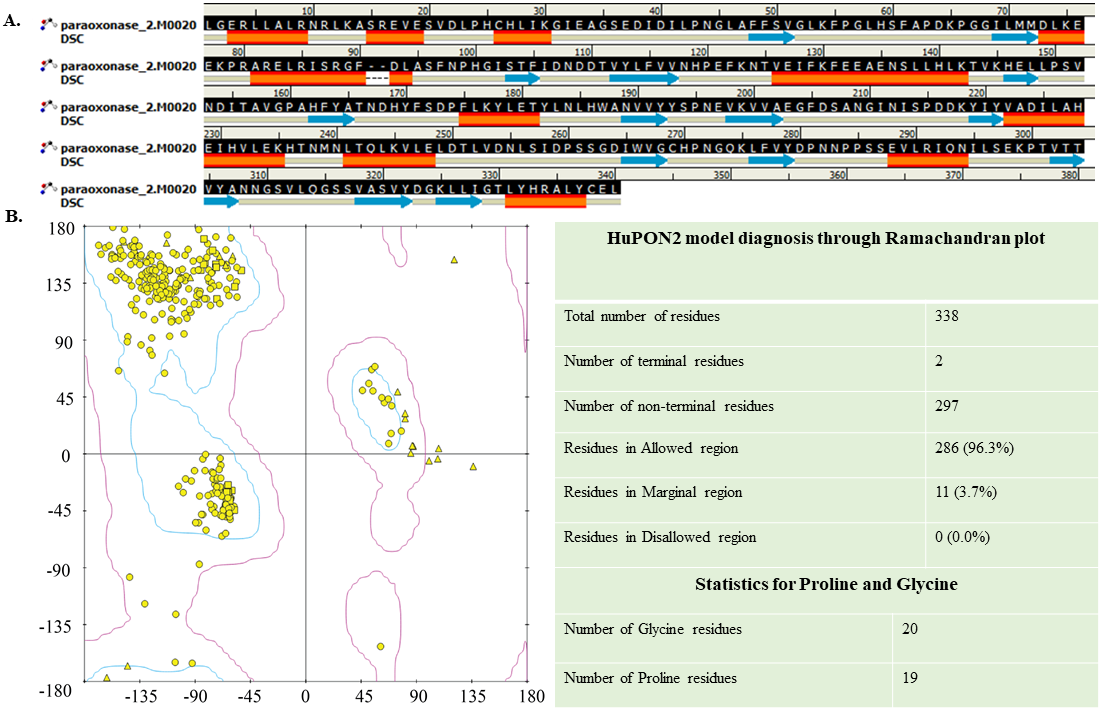

Supplement: S2 Fig — A. Predictive secondary structural analysis of the HuPON2 protein sequence. Orange and blue color beneath the primary sequence symbolizes the probability of alpha-helix and beta-sheet occurrence respectively. B. HuPON2 model was analyzed through Ramachandran plot. None of the residues were found in the disallowed region. (TIF) [file pone.0258879.s002.tif]

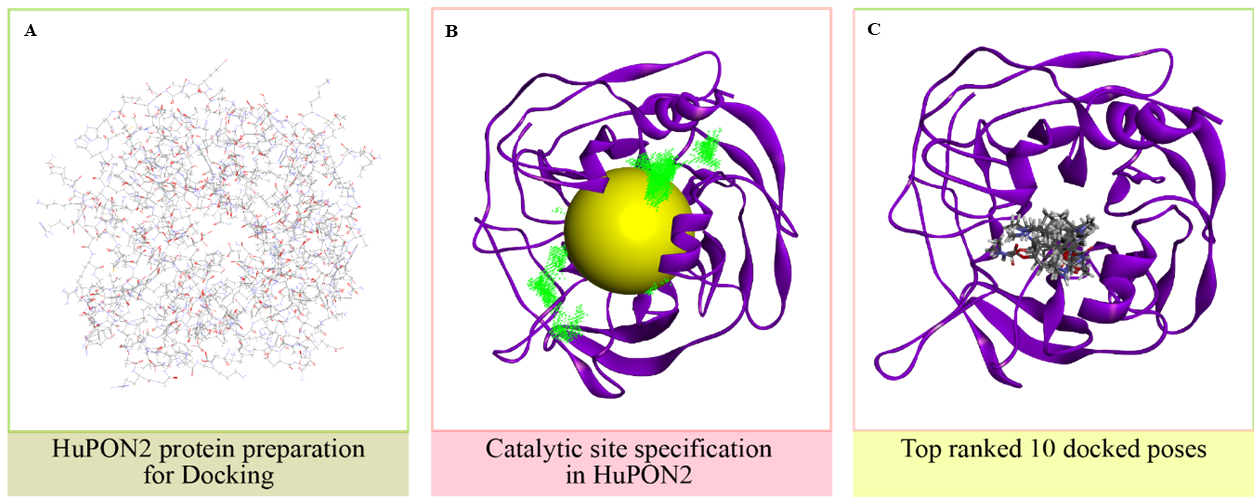

Supplement: S3 Fig — A. HuPON2 model protein was prepared for docking purpose, B. Catalytic site identification by yellow sphere ball. Green dots represent all the possible sites for drug docking, C. Best 10 docked poses of Eserine drug were filtered and represented in this panel. The docked pose is the first among the 10 times docking with different conformations of the protein. Discovery studio software was used for the visualization of the results and the creation of images. (TIF) [file pone.0258879.s003.tif]

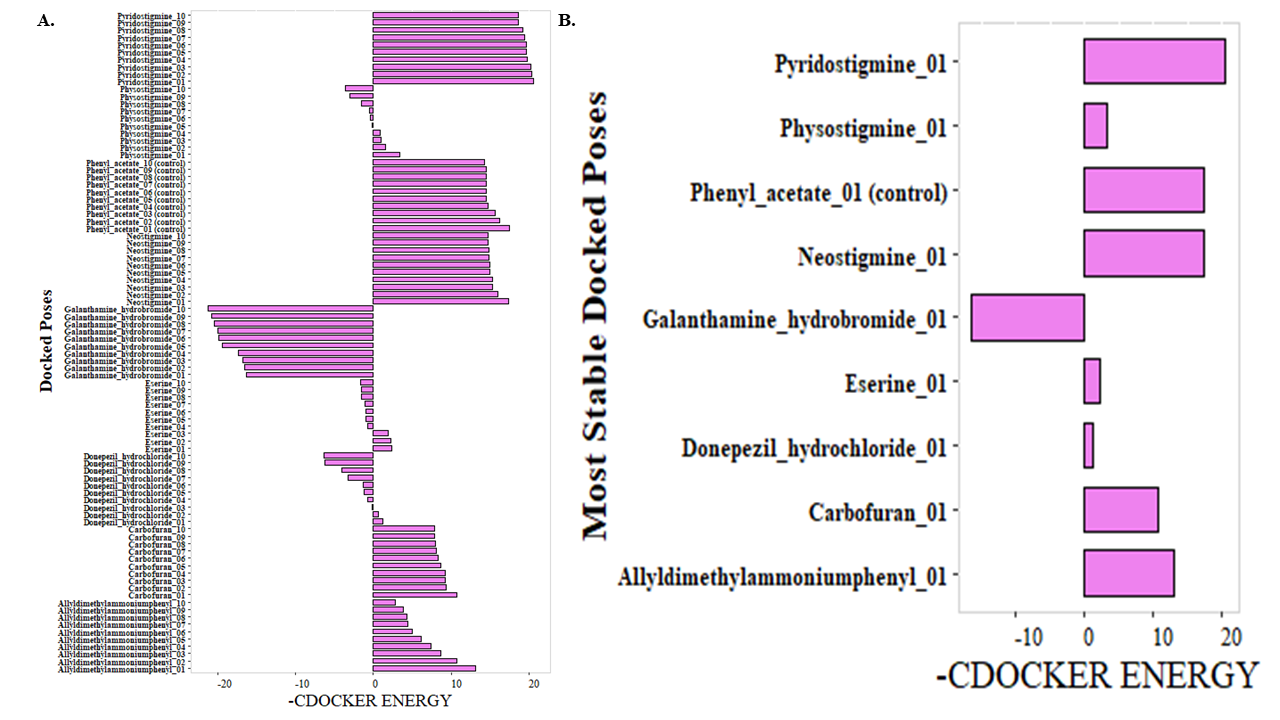

Supplement: S4 Fig — A. The docking library of all the screened drugs with HuPON2. B. The topmost stable conformations considered from the docking library were obtained. (TIF) [file pone.0258879.s004.tif]

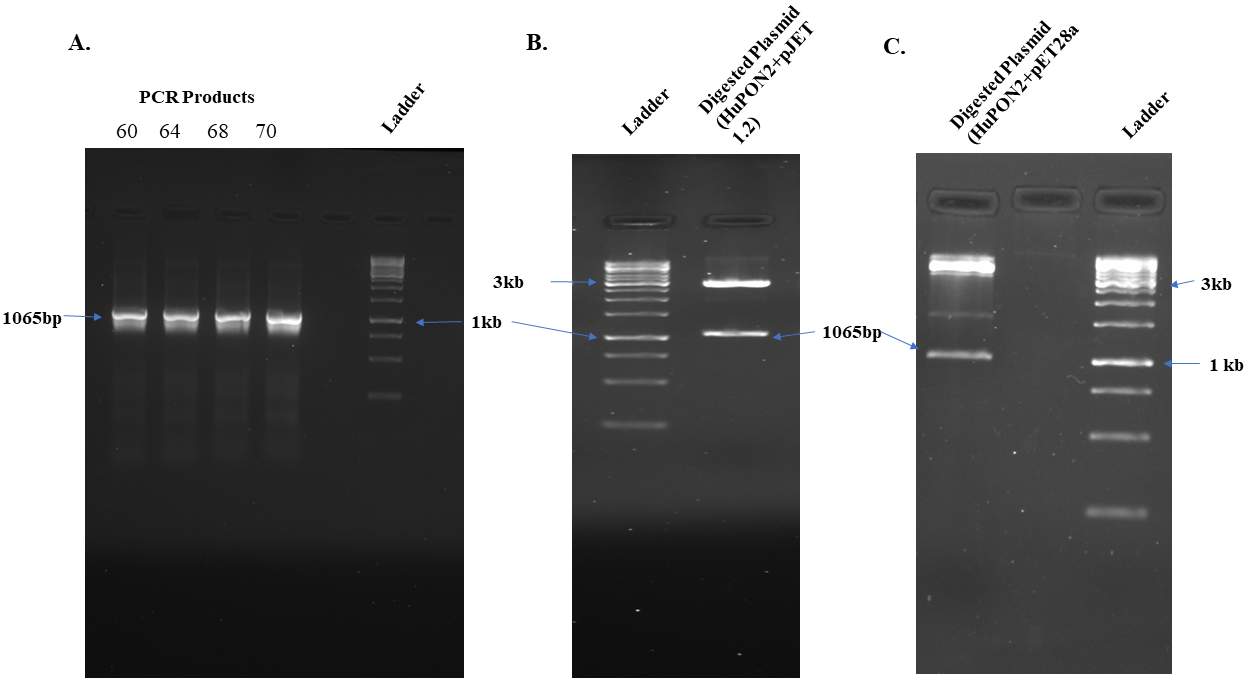

Supplement: S5 Fig — Agarose gel images showing, A. The PCR amplified product of HuPON2 gene, band size 1065 bp, gradient PCR was set at different temperatures (60֯C, 64֯C, 68֯C, 70֯C) B. confirmation of HuPON2 gene cloning in pJET1.2 vector by restriction digestion, C. confirmation of HuPON2 gene cloning in pET28a(+) vector by restriction digestion. (TIF) [file pone.0258879.s005.tif]

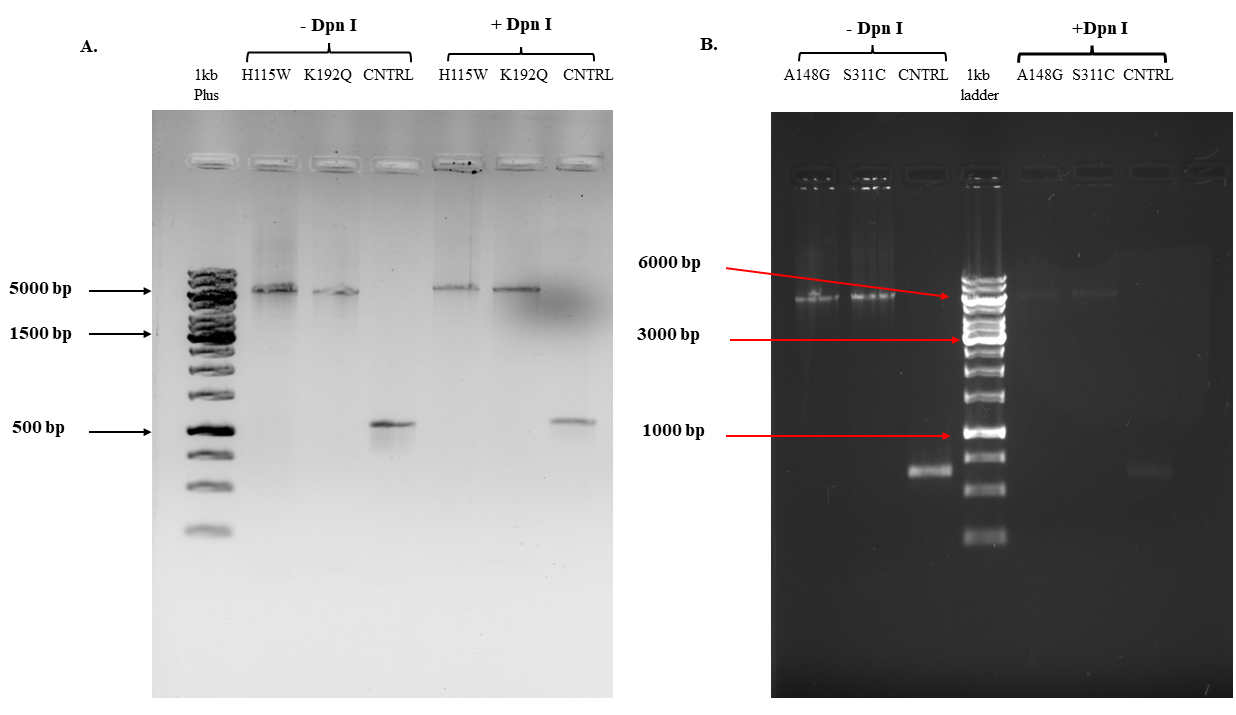

Supplement: S6 Fig — The efficiency of Dpn1 digestion of the PCR products is shown. The PCR products of plasmid pET 28(a)-HuPON2-WT with the indicated mutations, A. H115W, K192Q, and WT primer PCR control (CNTRL), B. A148G, S311C, and WT primer PCR control (CNTRL) were left untreated (-) or treated (+) with restriction enzyme Dpn1 and then analyzed by 1% agarose gel electrophoresis. Complete digestion of the WT plasmid can be seen in the control. (TIF) [file pone.0258879.s006.tif]

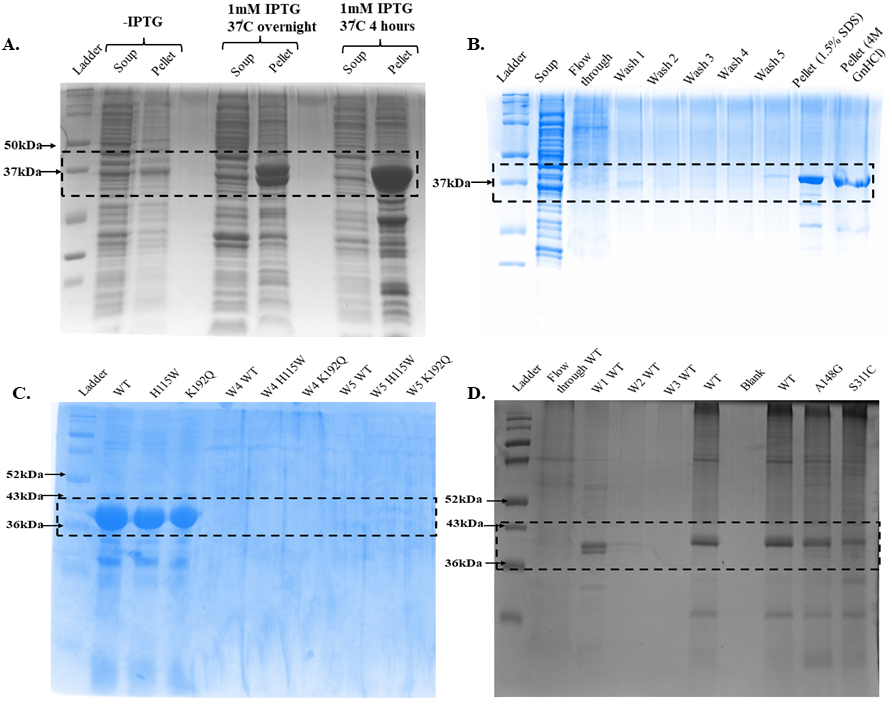

Supplement: S7 Fig — A. Expression at different temperatures showing most of the fractions in pellet, B. The soluble HuPON2 WT achieved by mild solubilization and denaturation, C. catalytic site mutant proteins solubilized in the same manner as WT and D. polymorphic site mutant proteins solubilized in the same manner as WT. (TIF) [file pone.0258879.s007.tif]

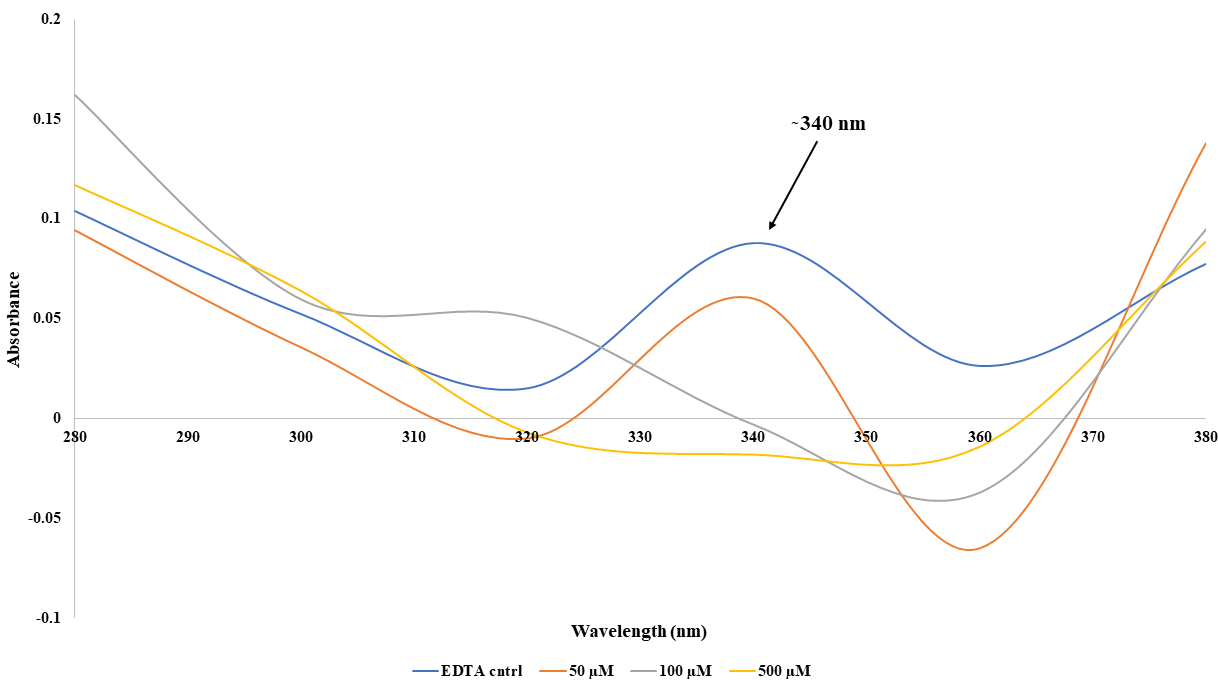

Supplement: S8 Fig — Different concentrations of Ca2+ ions were taken. UV-Visible data were acquired at 280–380 nm wavelengths. A concentration-dependent shift in the delta absorbance was observed at around 340 nm. (TIF) [file pone.0258879.s008.tif]
