# Supplementary material for: Association between human paraoxonase 2 protein and efficacy of acetylcholinesterase inhibiting drugs used against Alzheimer’s disease
Source: PLoS One. 2021 Oct 29;16(10):e0258879. doi: 10.1371/journal.pone.0258879 (PMC8555796; doi:10.1371/journal.pone.0258879)
Supplement: S1 Table — (DOCX) [file pone.0258879.s009.docx]

| **Name** | **Sequence** | **Tm** | **Restriction site** |
| --- | --- | --- | --- |
| **Cloning primers** | | | |
| HuPON2WT Fw | 5’ctaGCTAGCATGGGGCGGCTGGTGGCTGTG | 70 ֯C | Nhe I |
| HuPON2WT Rv | 5’ccgCTCGAGTTAGAGTTCACAATACAAGGCTCTGTGG | 65.5 ֯C | Xho I |
| **Mutant primers** | | | |
| *HuPON2-H115W* Fw | 5’ATTCAATCCA**TGG**GGCATCAGCACTTTCATAGA-3’ | 62.6 ֯C | - |
| *HuPON2-H115W* Rv | 5’GCTGATGCCCCATGGATTGAATGAGGCCAAAT-3’ | 66.1 ֯C | - |
| *HuPON2-K192Q* Fw | 5’GATCCTTTCTTA**CAG**TATTTAGAAACATACTTGAACTT-3’ | 57.1 ºC | - |
| *HuPON2-K192Q* Rv | 5’GTTTCTAAATACTGTAAGAAAGGATCAGAGAAGTAG-3’ | 56.6 ºC | - |
| *HuPON2-A148G* Fw | 5’AATTTGAAGAA**GGA**GAAAATTCTCTGTTGCATCTG-3’ | 64.8 ºC | - |
| *HuPON2-A148*G Rv | 5’CAGAGAATTTTCTCCTTCTTCAAATTTAAAAATTTC-3’ | 61.5 ºC | - |
| *HuPON2-S311C* Fw | 5’CAGAACATTCTA**TGT**GAGAAGCCTACAGTGACTAC-3’ | 68.3 ºC | - |
| *HuPON2-S311C* Rv | 5’GTAGGCTTCTCACATAGAATGTTCTGGATGCGGAG-3’ | 70.6 ºC | - |
