# Supplementary material for: Association between human paraoxonase 2 protein and efficacy of acetylcholinesterase inhibiting drugs used against Alzheimer’s disease
Source: PLoS One. 2021 Oct 29;16(10):e0258879. doi: 10.1371/journal.pone.0258879 (PMC8555796; doi:10.1371/journal.pone.0258879)
Supplement: S2 Table — The ranking is based on PDF total energy, PDF physical energy, and DOPE score. Among all these prepared models, the 20th number model is the best one. (DOCX) [file pone.0258879.s010.docx]

| **Model Scores** | | | |
| --- | --- | --- | --- |
| **Name** | **PDF Total Energy** | **PDF Physical Energy** | **DOPE Score** |
| Paraoxonase_2.M0020 | 2154.3918 | 1102.9974 | -40602.5391 |
| Paraoxonase_2.M0001 | 2232.4714 | 1111.7667 | -40566.1680 |
| Paraoxonase_2.M0002 | 2268.1836 | 1130.4193 | -40156.0313 |
| Paraoxonase_2.M0010 | 2371.3081 | 1134.3508 | -40369.8086 |
| Paraoxonase_2.M0007 | 2541.8521 | 1201.6643 | -40078.5703 |
| Paraoxonase_2.M0013 | 2567.3928 | 1223.5187 | -40149.3594 |
| Paraoxonase_2.M0015 | 2698.4175 | 1247.6146 | -39860.6641 |
| Paraoxonase_2.M0006 | 2703.1101 | 1306.5931 | -39083.6914 |
| Paraoxonase_2.M0003 | 2778.1873 | 1262.1842 | -39736.1328 |
| Paraoxonase_2.M0018 | 2795.2004 | 1275.1541 | -38909.5625 |
| Paraoxonase_2.M0014 | 2814.7598 | 1307.9556 | -38899.5625 |
| Paraoxonase_2.M0019 | 2822.9866 | 1275.1160 | -38913.2539 |
| Paraoxonase_2.M0009 | 2829.6909 | 1270.1285 | -38796.4023 |
| Paraoxonase_2.M0008 | 2850.5566 | 1302.8494 | -39185.4063 |
| Paraoxonase_2.M0016 | 2858.4146 | 1280.1688 | -38615.5391 |
| Paraoxonase_2.M0012 | 2901.0703 | 1318.1424 | -38870.5195 |
| Paraoxonase_2.M0011 | 2952.7983 | 1400.2470 | -38678.4180 |
| Paraoxonase_2.M0005 | 3441.8706 | 1580.0529 | -38384.7110 |
| Paraoxonase_2.M0017 | 3521.9338 | 1726.9346 | -38362.1523 |
| Paraoxonase_2.M0004 | 3710.0664 | 1878.1488 | -38239.6797 |
