# Supplementary material for: Association between human paraoxonase 2 protein and efficacy of acetylcholinesterase inhibiting drugs used against Alzheimer’s disease
Source: PLoS One. 2021 Oct 29;16(10):e0258879. doi: 10.1371/journal.pone.0258879 (PMC8555796; doi:10.1371/journal.pone.0258879)
Supplement: S3 Table — (DOCX) [file pone.0258879.s011.docx]

| **Drugs** | **CDOCKER_ENERGY** |
| --- | --- |
| Phenyl_acetate_01 (control) | 17.5138 |
| Eserine_01 | 2.3546 |
| Neostigmine_01 | 17.3954 |
| Physostigmine_01 | 3.3530 |
| Pyridostigmine_01 | 20.5979 |
| Allyldimethylammoniumphenyl_01 | 13.1286 |
| Galanthamine hydrobromide_01 | -16.2922 |
| Donepezil hydrochloride_01 | 1.1854 |
| Carbofuran_01 | 10.7588 |
